# Mental health provision in schools: approaches and interventions in 10 European countries

**DOI:** 10.1017/gmh.2017.6

**Published:** 2017-05-30

**Authors:** P. Patalay, D. Gondek, B. Moltrecht, L. Giese, C. Curtin, M. Stanković, N. Savka

**Affiliations:** 1 University College London, London, UK; 2 University of Liverpool, Liverpool, UK; 3 Maastricht University, Maastricht, The Netherlands; 4 University of Glasgow, Glasgow, UK; 5 Lund University, Lund, Sweden; 6 University of Limerick, Limerick, Ireland; 7 University of Niš, Niš, Serbia; 8 University of Sarajevo, Sarajevo, Bosnia & Herzegovina; 9 University of Warsaw, Warsaw, Poland

**Keywords:** Europe, intervention, mental health, prevention, promotion, school, support, well-being

## Abstract

**Background.:**

The role of schools in providing community-based support for children's mental health and well-being is widely accepted and encouraged. Research has mainly focused on designing and evaluating specific interventions and there is little data available regarding what provision is available, the focus and priorities of schools and the professionals involved in providing this support. The current study presents these data from schools in 10 European countries.

**Methods.:**

Online survey of 1466 schools in France, Germany, Ireland, Netherlands, Poland, Serbia, Spain, Sweden, UK and Ukraine. The participating countries were chosen based on their geographical spread, diversity of political and economic systems, and convenience in terms of access to the research group and presence of collaborators.

**Results.:**

Schools reported having more universal provision than targeted provision and there was greater reported focus on children who already have difficulties compared with prevention of problems and promotion of student well-being. The most common interventions implemented related to social and emotional skills development and anti-bullying programmes. Learning and educational support professionals were present in many schools with fewer schools reporting involvement of a clinical specialist. Responses varied by country with 7.4–33.5% between-country variation across study outcomes. Secondary schools reported less support for parents and more for staff compared with primary schools, with private schools also indicating more staff support. Schools in rural locations reported less student support and professionals involved than schools in urban locations.

**Conclusion.:**

The current study provides up-to-date and cross-country insight into the approaches, priorities and provision available for mental health support in schools; highlighting what schools prioritise in providing mental health support and where coverage of provision is lacking.

## Introduction

The prevalence of psychiatric disorders in school-aged children in European countries is generally estimated between 10% and 25% (Costello *et al.* 2003; Patel *et al.* 2007). Symptoms in childhood not only affect various domains of childhood development, but they are also antecedents to experiencing mental illness and further difficulties through the lifecourse (Roza *et al.* 2003; Copeland *et al.* 2015; Patalay *et al.* 2016*a*). There is support and recognition of the relevance of early and community-based intervention and health promotion in limiting the concurrent and lifelong ramifications of experiencing difficulties in childhood (Allen *et al.* 2014; Conti & Heckman, 2014).

Schools are considered a key setting for effective community-based mental health provision for many reasons, including their remit as educational institutions, access to young people and reduced stigma and increased inclusivity (Kavanagh *et al.* 2009; Greenberg, 2010; Caan *et al.* 2014). Schools already work towards well-being oriented goals, such as building friendships or developing self-identity whilst being able to rely on extensive supportive networks of their students comprising of peers, teaching staff, other professionals and parents (Jané-Llopis & Braddick, 2008). Moreover, schools have day-to-day contact with young people, and often their families, allowing for effective screening for problems and implementing both universal and targeted interventions (Stephan *et al.* 2007; Jané-Llopis & Braddick, 2008; Caan *et al.* 2014).

The role of schools in providing mental health support has been increasingly prioritised, although to a different extent in various countries (Weare & Nind, 2011). At the European level, the importance of schools in supporting mental health of children and young people has increasingly been reflected in recent European level initiatives and policies. Of note, the Child and Adolescent Mental Health in Enlarged European Union (CAMHEE) project, aimed to provide opportunities for knowledge exchange and learning between European states to support greater evidence-based practice. Within this initiative, experts from different European countries have gathered information on available policies, existing programmes, workforce and infrastructures for mental health treatment and promotion in each country (Braddick *et al.* 2009; Puras & Sumskiene, 2009). However, data directly from schools are not available on approaches to mental health and existing provision. Another relevant policy action at the EU level is the current European Joint Action on Mental Health and Wellbeing (2013–2016). The Action facilitates cooperation between Member States, relevant stakeholders, international organisations and EU institutions from 30 European countries. One of the key issues it addresses is the promotion of mental health in schools. Its work packages include ‘developing community-based and socially inclusive approaches’ and ‘promoting cooperation across education, health and social sectors in mental illness prevention amongst children and adolescents’ (http://www.esn-eu.org/european-joint-action-on-mental-health-and-well-being/index.html). The European Union Dataprev project confirms that over the past 25 years there has been a significant increase in the number of large scale school mental health programmes (Weare & Nind, 2011).

In terms of school-based programmes and interventions for promoting student well-being and supporting students with difficulties, there are hundreds of studies and trials investigating the efficacy and impact of these programmes (Green *et al.* 2005; Adi *et al.* 2007; Tennant *et al.* 2007; Wolpert *et al.* 2015). Although, the literature investigating the development and effectiveness of mental health provision in schools has been growing, very little is known about what schools actually do currently to support student mental health (NHS England & Department of Health, 2015). We identified only two existing studies that have used survey methodology to map out existing provision in schools for supporting student mental health. Vostanis *et al.* (2013) examined the nature and level of prescriptiveness of approaches taken by primary and secondary schools in England based on a survey in 2009. They found that the interventions were mostly reactive, that is, addressing pupils with already existing problems, whereas preventative and promotional approaches were less widespread. Moreover, individuals involved in mental health provision tended to lack any specialist training. The most frequently used strategies in mental health support included social and emotional skills development, creative and physical activities and learning and structural support (Vostanis *et al.* 2013). In the USA, Teich *et al.* (2008) investigated what types of mental health problems are encountered in schools and how these are addressed. Inspired by these two studies and recognising the need for more information on existing provision in schools, especially in the European context, the current study presents data from schools in 10 European countries regarding their existing mental health provision. We examine this both in terms of the approaches taken to support within schools (i.e. universal/targeted; treatment/promotion) and by examining the existing provision in schools (specific programmes/interventions and professionals involved).

## Methods

### Sampling

The aim of the research group was to obtain data from a diverse range of European countries that covered a geographical and economic spread. In line with this aim, selected countries belong to different geographical parts of Europe (Sweden from Scandinavia, Spain from the south-west, Germany and Poland from central Europe and Ukraine from Eastern Europe, etc.), not all are EU members (e.g. Ukraine and Serbia) and they represent diverse political and economic systems. The final countries included in the study were also selected based on convenience in terms of access to the research group and presence of collaborators, hence resulting in the 10 countries present in this study.

### Participants

Participants were 1466 schools from 10 European countries [France: *n* = 80 (5.5%), Germany: *n* = 194 (13.2%), Ireland: *n* = 171 (11.7%), Netherlands: *n* = 148 (10.1%), Poland: *n* = 225 (15.4%), Serbia: *n* = 222 (15.1%), Spain: *n* = 87 (5.9%), Sweden: *n* = 44 (3.0%), UK: *n* = 181 (12.4%), Ukraine: *n* = 114 (7.8%)]. Of the schools that participated, 52.1% (*n* = 764) were primary schools, 34.6% (*n* = 507) were secondary schools, 11.3% (*n* = 166) were combined primary and secondary schools and 1.9% (*n* = 28) were classified as other (e.g. pre-schools); 92.1% (*n* = 1350) of schools were state funded and 7.9% (*n* = 116) were privately funded. In addition, 57.3% (*n* = 840) of schools stated their location as being urban and 42.7% (*n* = 626) as rural. Average school size was 436.66 students (s.d. = 459.43). The majority of school staff that answered the survey were head teachers (*n* = 673; 45.9%), followed by teachers (*n* = 311; 21.2%), school psychologists (*n* = 185; 12.6%) and deputy head teachers (*n* = 183; 12.5%).

### Procedure

On the basis that the project does not include personal information, it was deemed unnecessary by the institutional ethics committee to undertake a full research committee review. The email addresses of schools were acquired through engaging with educational departments and through accessing online databases. Following this, schools were sent an email requesting them to identify the appropriate individuals to complete the survey for their school [‘We request you to identify person(s) best suited to answer questions regarding current provisions and interventions to support mental health and well-being in your school to complete the survey’]. Schools were provided with a link to the survey and were informed about the confidentiality of individual school responses within the email. Having accessed the survey, schools were given further information about the study and consented to participating before completing the survey. All data were collected within the academic year from September 2013 to June 2014.

### Measures

The measure was developed based on the existing research (Teich *et al.* 2008; Wolpert *et al.* 2011; Vostanis *et al.* 2013) and through liaising with researchers and school staff (greater details of the measure development and content are available – Patalay *et al.* 2014). Initially, based on the existing literature and the aims of the study, the research group, comprising all authors of the manuscript, determined the key areas of focus for the survey with input from advisors (which included researchers, school staff and clinical and educational psychologists from each of the respective countries). The measure was developed in a constant cycle of question development, translation and focus groups/interviews in participating countries, which fed back into question development. Hence, although the master version of the questionnaire was maintained in English, feedback from teachers/psychologists across the participating countries shaped the content and language of the survey. Focus groups, interviews and pilot surveys were carried out with teachers and psychologists, recruited through convenience-based strategies, to ensure overall reliability of the survey, including appropriate interpretation, coherence and optimal understanding of the questions translated into different languages in the online surveys. The survey content, order, formatting and presentation was consistent across translations. The survey items and response options in the English version of the online questionnaire can be found in the online Supplementary Table 1. For versions in the other languages (German, Dutch, Spanish, French, Polish, Ukrainian and Serbian) please contact the corresponding author. The survey first included some preliminary descriptive questions about the school (country, primary/secondary, state/private funding, location and size). The subsequent items corresponded to the approaches and interventions the schools adopt towards mental health provision and the personnel involved in this provision in the school.

#### Approach to mental health provision in schools

The school's approach to mental health support was captured through questions regarding the extent of their focus: (1) the focus and reach of the support provided (universal and targeted) and (2) the target group for support (children with identified mental health problems, children with learning disabilities, children starting to develop problems, preventing problems from arising and proactively promoting well-being). For each of these items, schools responded on a 5-point scale ranging from ‘not at all’ to ‘very much’.

#### Existing provision in schools

To assess the existing interventions in schools, participating schools were presented with a list of interventions and supports for students, parents and staff and asked to select to what extent the school implemented such initiatives (‘not at all’ to ‘very much’). The list included a diverse range of options, including social and emotional skills development programmes, individual and group therapy, anti-bullying programmes and mental health education (the full list can be viewed in Table 2). For parents/carers we asked about the presence of information, training, counselling and support. In addition, for teachers we also included supervision and consultation with mental health professionals and a well-being programme directed at teachers themselves (items in Table 3). Subsequently, schools were asked to indicate which professionals were typically involved in mental health and well-being provision in their school [options included school nurse, school psychologist, learning/special educational needs (SEN) support, social worker, clinical psychologist/psychiatrist and an ‘other’ option under which schools could specify other professionals that had not been listed].

### Analysis

In accordance with the aims of the current study we first investigate the approaches towards school mental health provision overall, followed by a country-level investigation of differences in approaches taken by schools. Subsequently, we examine existing mental health provision (the range of different interventions and the professionals involved) by presenting descriptive statistics indicating overall response levels and the between country-level variation [intraclass correlation coefficient (ICC)]. Then we examine the school characteristics (type, funding and location) that predict variation in the study outcomes, by conducting a multi-level (to account for schools being nested within countries) regression analysis predicting overall levels of intervention available for students, their parents and school staff and the professionals involved. For all outcomes we present data graphically for the 10 participating countries to demonstrate country-level differences in school provision.

## Results

### Approaches to mental health provision in schools

Results in Table 1 demonstrate that in their approach to mental-health and well-being provision, substantially schools focus ‘quite a lot’ or ‘very much’ on the whole school (universal approach; 68.8%) as opposed to targeting individuals with specific problems (51.3%).

**Table 1.** Approaches to mental health support in schools

		Not at all (%)	A little (%)	Somewhat (%)	Quite a lot (%)	Very much (%)	Average score	% of country variance (%)
Target group								
Treatment	Children with learning disabilities	1.1	5.0	15.9	47.8	30.2	4.01	14.70
	Children with already identified mental health problems	2.3	8.9	22.1	45.4	21.2	3.74	8.18
Prevention	Children starting to develop problems	3.5	9.8	20.6	43.6	22.6	3.72	7.39
	Preventing problems from arising	4.4	12.6	28.0	40.3	14.8	3.49	17.40
Promotion	Pro-actively promoting well-being	5.3	15.1	29.5	36.8	13.3	3.38	19.63
Focus and reach								
Universal/whole school		1.9	8.3	20.9	43.8	25.0	3.82	12.55
Targeted		5.3	14.9	28.4	35.8	15.5	3.41	13.51

**Fig. 1.**
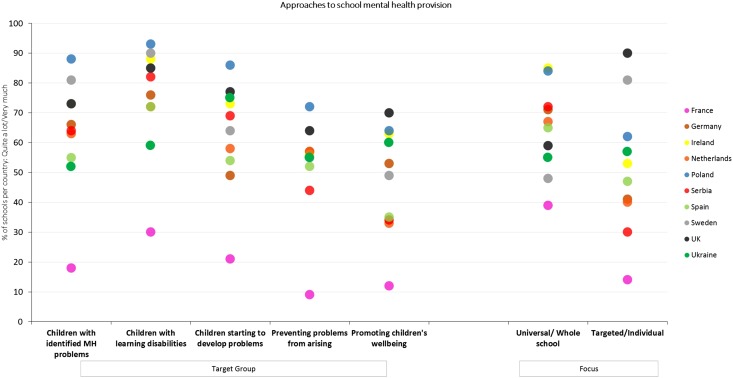
Demonstrates the country-level variation in the target group of provision and the focus and reach of the approaches that schools take across the 10 participating European countries.

Figure 1 includes the country-level response estimates (as a percentage of schools) for the target group and focus of the provision that is available in all 10 participating countries. As shown in Fig. 1, the percentage of schools focusing ‘quite a lot’ or ‘very much’ on a whole school approach was highest in Ireland (85.4%) and Poland (83.7%) and lowest in France (38.7%), whereas the percentage in other participating countries ranged from 47.6% to 72.0%. The targeted approach appeared to be the main focus in the UK (89.8%) and Sweden (81.0%). Again, the lowest percentage of schools focusing ‘quite a lot’ or ‘very much’ on individual students was found in France (13.7%), with other participating countries ranging from 30.0% to 61.6%. The between country-level variance was found to be very similar for both approaches, with 12.6% for the universal and 13.5% for the targeted approach.

In terms of the focus of provision, the provision tends to be treatment-oriented (see Table 1), with schools indicating most support (‘quite a lot’ or ‘very much’) for children with learning disabilities (78.0%) and children with already identified or developing mental health problems (66.6% and 66.2%). A further 55.1% of schools focused ‘quite a lot’ or ‘very much’ on preventing problems from arising. Finally, half of the schools (50.1%) indicated that they focus ‘quite a lot’ or ‘very much’ on pro-actively promoting well-being amongst their students.

In terms of country-specific results, Poland and Sweden had the highest percentage of schools that reported focusing ‘quite a lot’ or ‘very much’ on supporting children with learning disabilities, 92.9% and 90.5% respectively, whereas the lowest percentages were noted in Ukraine (58.8%) and France (30.3%). The percentages for other countries ranged from 72.0% to 87.7%. Children with identified mental health problems were the main focus in Poland (88.2%) and Sweden (81.0%), whereas the lowest percentage of schools reporting to focus on these students was in France (18.7%). The percentage for other participating countries ranged from 52.0% to 72.9% (see Fig. 1).

The preventative approach, focused on children starting to develop problems, was most prominent in Poland, with 85.8% of schools implementing it ‘quite a lot’ or ‘very much’. In comparison, only 21.3% of French schools focus on this approach ‘quite a lot’ or ‘very much’, with other participating countries, ranging from 49.2% to 71.6%. Similar results were found regarding the extent to which schools focus on preventing mental health problems from arising, where Poland was again the country with the highest percentage of schools responding ‘quite a lot’ or ‘very much’ (71.6%) and France had the lowest percentage of schools giving such a response (9.3%). The percentage for other participating countries ranged from 44.2% to 63.8% (see Fig. 1). Finally, proactive promotion of well-being was the main focus in 70.1% of schools in the UK, which was the highest percentage, and only in 12.2% in France, having the lowest percentage of schools focusing on such approach ‘quite a lot’ or ‘very much’. The percentage for other participating countries ranged from 32.6% to 63.5%.

The variance between countries was observed to be lowest for the approach focusing on children starting to develop problems (7.4%) and children with already identified problems (8.2%), whereas the highest variance was reported for approaches that aim at preventing problems from arising (17.4%) and pro-actively promoting well-being (19.6%).

### Existing provision in schools

As presented in Table 2, schools implement mostly physical activities (73.6% of schools indicating ‘quite a lot’ or ‘very much’), behaviour support (65.5%), creative activities (65.4%) and social skills development (60.9%) in order to support mental health and well-being of their students. On the other hand, the least used interventions to support students are mindfulness (8.5% of schools indicating ‘quite a lot’ or ‘very much’), having a designated space for well-being/mental health support (16.2%), group therapy (16.3%) and mental health education (16.8%).

**Table 2.** Interventions supporting students’ mental health

	Not at all (%)	A little (%)	Somewhat (%)	Quite a lot (%)	Very much (%)	Average score	Country-level variance (%)
Social skills development	2.1	8.9	28.1	44.2	16.7	3.64	12.69
Emotional skills development	3.8	15.3	34.1	36.4	10.4	3.34	23.49
Creative activities	1.0	8.3	25.3	41.1	24.3	3.79	19.73
Physical activities	1.0	4.2	21.2	44.0	29.6	3.97	9.42
Signposting	4.7	19.5	37.5	28.8	9.5	3.19	13.17
Peer support	6.2	19.4	35.2	30.1	9.1	3.17	10.02
Behaviour support	2.6	6.5	25.4	43.3	22.2	3.76	16.47
Designated space for well-being/mental health support	40.3	23.6	19.9	10.2	6.0	2.18	18.24
Infrastructure for extra-curricular activities	7.8	19.8	29.5	27.6	15.3	3.23	10.80
Individual therapy	22.4	21.5	24.8	22.4	8.9	2.74	13.56
Group therapy	35.5	23.7	24.5	13.0	3.3	2.25	21.90
Mindfulness	39.1	32.5	19.9	6.8	1.7	1.99	11.46
Anti-bullying programme	9.3	12.1	25.4	32.8	20.4	3.43	28.01
Risky health behaviour programme	6.8	13.5	28.9	36.3	14.5	3.38	19.98
Mental health education	26.9	27.2	29.1	13.4	3.4	2.39	15.49

**Fig. 2.**
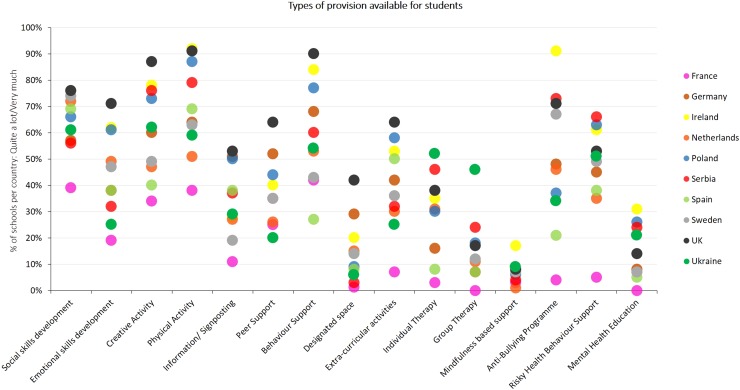
Demonstrates the country-level variation in the different interventions available in schools across the 10 participating European countries.

**Fig. 3.**
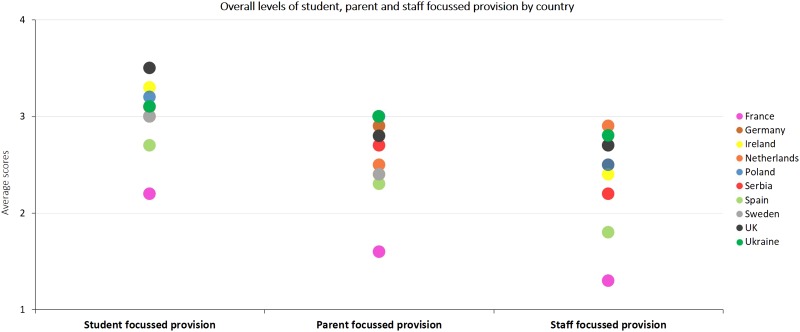
Demonstrates the country-level variation in the total amounts of student, staff and parent focused support available in schools across the 10 participating European countries.

Figures 2 and 3 contain country-level estimates (as a percentage of schools in each country) for the range of support provision available for pupils, staff and parents in the 10 participating countries. As observed in Fig. 2, the country-level variance was highest for anti-bullying programmes (28.0%), emotional skills development (23.5%) and group therapy (21.9%), whereas the lowest country-level variance was observed for physical activities (9.4%), peer support (10.0%) and infrastructure for extra-curricular activities (10.8%).

Table 3 presents the extent to which schools provided support for parents and staff which might enable them to better support student mental health. Schools mainly provided information for parents and a fifth of schools also provided parents with counselling and support. Around a quarter of schools reported that they provide training and education for staff regarding mental health and fewer schools (<20%) provided supervision, counselling and support for staff well-being.

**Table 3.** Support available for parents and staff in schools

	Not at all (%)	A little (%)	Somewhat (%)	Quite a lot (%)	Very much (%)	Average score	Country-level variance (%)
Parents							
Information	7.2	18.5	33.5	31.9	9.0	3.17	17.73
Training	29.3	27.6	28.8	12.2	2.2	2.31	11.22
Counselling and support	26.9	25.2	26.4	16.5	5.0	2.48	25.56
Staff							
Training and education	16.7	23.5	34.5	20.4	4.9	2.73	11.61
Supervision and consultation	29.7	23.9	27.9	15.3	3.2	2.38	8.01
Counselling and support	26.2	29.7	29.0	12.5	2.6	2.36	18.56
Well-being programme	30.4	26.7	30.5	10.1	2.3	2.27	33.46

As seen in Fig. 3, schools in the UK and Ireland indicated having the highest levels of interventions directed at students (3.47 and 3.34 overall score, respectively; overall score represents an average across all interventions available), whereas Ukraine and Germany indicated most support for parents and the Netherlands had most support for staff. The lowest levels of interventions across the board were observed in France, followed by Spain. Between country variations in overall amount of support provided ranged from 18% to 27% (ICCs are presented in Table 4), indicating substantial country-level differences in amount of provision for students, parents and staff.

**Table 4.** School characteristics predicting existing provision for students, parents and staff and the professionals involved in mental health support

	Student provision Coef (SE)	Parent provision Coef (SE)	Staff provision Coef (SE)	Professionals involved Coef (SE)
School type^a^ (secondary)	0.08* (0.03)	−0.17*** (0.05)	−0.02 (0.05)	0.02 (0.01)
School type^a^ (primary and secondary)	0.25*** (0.07)	0.06 (0.11)	0.32** (0.11)	0.03 (0.03)
School type^a^ (other)	−0.22 (0.12)	−0.77*** (0.18)	0.14 (0.17)	0.02 (0.05)
School funding^b^ (private)	0.02 (0.06)	0.16 (0.09)	0.18* (0.09)	−0.02 (0.02)
School location^c^ (rural)	−0.10*** (0.03)	−0.19*** (0.05)	−0.20 (0.05)	−0.03* (0.01)
Variance estimates				
Country-level	0.34 (0.08)	0.39 (0.09)	0.44 (0.10)	0.11 (0.02)
Residual	0.56 (0.01)	0.82 (0.02)	0.82 (0.02)	0.24 (0.00)
ICC	0.27 (0.09)	0.18 (0.07)	0.22 (0.08)	0.16 (0.06)

a School type, reference category primary schools.

b School funding, reference state-funded.

c School location, reference urban.

* < 0.05; ** < 0.01; *** < 0.001.

**Fig. 4.**
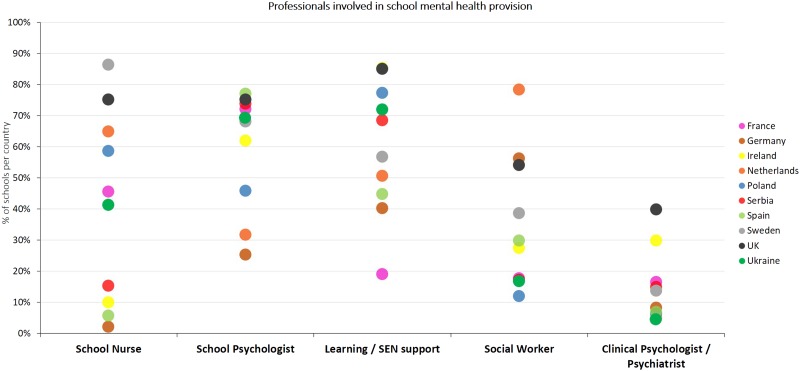
Demonstrates the professionals involved in support of mental health of pupils at schools across the 10 participating European countries.

In terms of professionals involved in mental health provision, those most commonly listed were learning or SEN support (64.2%) and school psychologists (57.2%), whereas the involvement of other professionals was reported to a lesser extent, e.g. school nurses (37.2%), social workers (34.9%) and clinical psychologists/psychiatrists (15.2%). One-third of the respondents (34.7%) indicated that other professionals not mentioned in the survey options, also tend to be involved in provision such as school counsellors or behaviour support staff in the UK, and support teachers and nursery teachers in Germany. Figure 4 presents the percentage of schools per country that report having a range of professionals (such as nurses and psychologists) involved in providing mental health support in their schools.

Country-level differences (see Fig. 4) indicate that the involvement of particular professionals across participating countries varied greatly. For instance, school nurses are most involved in Sweden (86.4%) and the UK (75.1%), whereas their involvement is least in Spain (5.7%) and Germany (2.1%). School psychologists are involved to the greatest extent in schools in Spain (77.0%), UK (75.1%), Serbia (73.9%) and France (72.2%), whereas only 25.3% of schools in Germany report their involvement. Learning and SEN support staff are more prevalent in Ireland (85.4%) and the UK (85.1%), whereas in France only 19% of schools indicated involvement of these professionals. Social workers were indicated to support mental health/well-being of students mostly in the Netherlands (78.4%), and least commonly in Poland (12.0%), Ukraine (16.7%), Serbia (17.1%) and France (17.7%). Finally, clinical psychologists are more involved in school-based mental health provision in the UK (39.8%) and least in Ukraine (4.4%), the Netherlands (4.7%), Poland (6.2%) and Spain (6.9%)

In terms of school-level predictors of existing provision (regression results in Table 4), secondary schools reported greater mental health provision for students and were significantly less likely to support parents compared with primary schools. Schools that accommodated both primary and secondary school aged year groups also reported greater pupil and staff provision compared with primary schools only. School funding did not predict the amount of pupil provision. However, private schools were more likely to provide support and training to staff. School type and school funding did not predict the extent of involvement of various professionals in mental health support. Finally, school location was a significant predictor of mental health support for students, parents and staff, with urban schools having greater mental health provision. Urban schools were also more likely to have a greater range of professionals involved in mental health support.

## Discussion

This study maps the current approaches to school mental health provision in a range of European countries, including specific activities and professionals supporting mental health in schools. Moreover, we examined school characteristics (e.g. primary/secondary, funding and location) that might predict differences in extent of current provision. Being the first such study conducted across numerous European countries, our findings also provide a baseline for future developments in school mental health; for instance, the impact of current policies such as the European Joint Action on Mental Health and Wellbeing (2013–2016) on school-based mental health provision in coming years.

The focus on Europe in the current study is also important. Studies examining the development and efficacy of interventions are predominantly from the USA (Weare & Nind, 2011). Given the increased policy focus at the European level on child mental health and the role of schools in providing support, data on what schools are doing at present is an important benchmark to understand developments in available support in the coming years.

In terms of how schools approach mental health support for their students, we found that on average schools indicated a greater focus on universal approaches compared with targeted approaches. Interestingly, at the country level there was greater variation between countries for targeted approaches with high numbers of schools in the UK (90%) and Sweden (81%), reporting focusing on targeted approaches, compared with only 14% in France. In contrast in France, 38% of schools reported universal approaches to supporting their students’ mental health. Universal approaches were least prevalent in France (38%) and most prominent in Poland and Ireland with over 80% of schools reporting a focus on universal provision. Similar to findings from Vostanis *et al.* (2013) in England, schools across the surveyed countries in Europe implemented interventions that are mostly reactive with respect to existing or emerging mental health problems, while they focused less on preventative and promotional approaches. For instance, overall, around 66% of schools reported focusing ‘quite a lot’ or ‘very much’ on children with already identified mental health problems and 78% on children with learning difficulties. In contrast, around half the surveyed schools focused on preventing problems and promoting student well-being. This focus on preventative approaches, on average, is similar to the 63% reported by Teich *et al*. (2008) in the USA; however, there is large variation (21–86%) between the surveyed countries in our study. Variation between countries in the focus of school mental health provision was highest for promotion with 20% of variation explained by differences between countries. At a country level, our data indicated that schools in the UK (70%) had the greatest focus on proactive promotion of well-being compared with 11% of French and 33% of Dutch schools.

The most frequently used strategies in mental health support included social skills development programmes, behaviour support, and creative activities; which are strategies that have been implemented in schools traditionally for years. These curriculum and classroom-based approaches to enhance social and emotional functioning have been found to be most commonly cited as being effective (Teich *et al.* 2008). Less traditional strategies such as mindfulness practices and the use of designated spaces for well-being have also made their way to schools across Europe, however to a much lesser extent. As the evidence base for these more recent strategies develops (Kuyken *et al.* 2013), it can be expected that in future years such interventions might become more widespread, which is something future research can investigate using these data for comparison.

The types of professionals involved in the mental health provision varied across countries. This may be a reflection of the different foci and policies present in these countries. For instance, in Sweden it is obligatory for schools to have a school nurse and this is reflected in the 86% who indicated that school nurses play a role in supporting the mental health of their students. In a recent report, the English Department for Education indicated that more than 80% of schools have access to a trained school counsellor (Department for Education, 2015), which is reflected in the UK having the highest proportions of schools stating that counsellors (75%) and clinical psychologists/psychiatrists (40%) are involved in mental health provision within their schools.

In terms of school characteristics that predict extent of available provision in schools, we found that schools including adolescents (secondary and combined primary/secondary) reported higher levels of pupil targeted support compared with primary schools, possibly reflecting the greater need and symptom prevalence during adolescence (Costello *et al.* 2011). Our results indicated that there were no significant differences in mental health provision for students based on school funding. However, private schools were more likely to provide support and training to staff than public schools. Provision and training for staff and parents have been widely neglected within schools (Vostanis *et al.* 2013). In light of the role parents and teachers play in screening and early recognition of difficulties, increased training, information and support for staff may have positive downstream effects and should hence be encouraged (Jorm *et al.* 2010). School location also predicted the extent of available provision, with schools in rural locations reporting significantly lower amounts of provision and lesser extent of professionals involved in delivery. Similar results have been reported in the USA whereby urban schools were more likely to have arrangements or other formal agreements with community-based mental health organisations than rural schools (Teich *et al.* 2008). The lack of access to specialist staff is identified as a key barrier to sufficient mental health provision by schools (Patalay *et al.* 2016*b*), and our findings indicate that schools in rural areas might be disproportionately affected by the limited access to trained and specialist staff.

A striking country-level observation is the lower levels of available provision in France. Schools in France consistently reported much lower levels of provision, although in terms of professionals involved schools reported the presence of a variety of professionals who support student mental health. This finding could reflect the more curriculum- and academic outcomes oriented focus of French schools (Gumbel, 2010). It is also possible that schools in France underestimate the support that they are providing, which in fact may be similar to some of the other countries included in the study.

Overall, at a national level, no countries focused on the whole range of approaches (e.g. high levels of targeted and universal approaches), although increasingly there is support for universal approaches combined with targeted approaches as being the most effective strategy for providing support in schools (Banerjee *et al.* 2016). This is also observed when considering approaches that are focused on reactive treatment, prevention and promotion approaches, with few countries having high levels of provision in all these categories (Poland and the UK report somewhat high levels across categories and France reports low levels across all of them). These data highlight that there might not be a gold standard yet, at a national level, for schools providing sufficient and suitable support for all their students – and these are highlighted in their reporting of key barriers to provision, including availability of specialists, funding and staff capacity (Patalay *et al.* 2016*b*). At a European level, strategies might be devised and learning shared between countries under the auspices of programmes such as the European Joint Action on Mental Health and Wellbeing, which can be informed by the results of studies such as these.

The current study has several strengths, including a comprehensive school survey, coverage of 10 European countries and filling in a much-needed gap in the literature. Nonetheless, the study is not free from limitations. Although we attempted to reduce selection biases by inviting all schools to participate for whom contact details were available, it is likely that schools with an interest in mental health might have been more likely to complete the survey leading to a possible overestimation of existing provision. It is also important to note that the responses are based on school staff's reports of available resources and might be limited by the knowledge that the particular staff member(s) that were selected to complete the survey for their school. In a similar vein, completion of the survey by different professionals may have resulted in a response bias, as they may have different level of knowledge of different aspects of the provision. For instance, deputy head teachers may be better informed about existing school or national policies, whereas school psychologists can possibly have a greater knowledge of specific interventions aiming at improving mental health or well-being at the school. In future, such data might be complemented with more objective reports based on observations, budget and resource allocation to help provide a more accurate picture of existing provision. It would be particularly beneficial to obtain more information on the process of accessing more specialised mental health support, as the present study focused simply on availability of such provision. For instance, future research should explore what the referral system looks like, which professional groups children are more likely to be referred to in each country, what the waiting time is and if the services are free of charge for students.

In conclusion, the current study provides much needed overview of the existing approaches to and provision for mental health and well-being support of students across the 10 participating countries in Europe. This serves as a useful benchmark against which the impact of recent policies at European and national levels can be assessed. Finally, through cross-national comparisons our study provides information that can facilitate knowledge exchange and experience sharing between countries with the aim of having adequate school-based mental health provision across Europe.

## Supplementary material

For supplementary material accompanying this paper visit http://dx.doi.org/10.1017/gmh.2017.6. click here to view supplementary material
