# Supplementary material for: Mental health provision in schools: approaches and interventions in 10 European countries
Source: Glob Ment Health (Camb). 2017 May 30;4:e10. doi: 10.1017/gmh.2017.6 (PMC5454766; doi:10.1017/gmh.2017.6)
Supplement: Supplementary file 1 [file S2054425117000061sup001.docx]

*Supplementary material*

**Mental health provision in schools: Approaches and interventions in 10 European countries**

Patalay, P., Gondek, D., Moltrecht, B., Giese, L., Curtin, C., Stanković, M., Savka, N.

In this supplementary file we present the sections of the online survey that are presented in this paper below (English version).

For the full questionnaire used in the study and the versions in different languages please contact the corresponding author.

| *--------section break in online survey-----------* |
| --- |
| Country: |
| School type: Primary/Secondary |
| Type of school funding: State funded/Private |
| Location: Urban/rural |
| Number of students: _______________________ |
| Number of teaching staff: ___________________ |
| Average class size: _________________________ |
| *--------section break in online survey-----------* |
| This section concerns types of interventions your school provides to support the mental health and well-being of students, their parents/carers and school staff. |
| **To what extent does your school do the following activities?** |
| **For Students:** (**not at all/ a little/ somewhat/ quite a lot/ very much)** |
| Social skills development (e.g. interpersonal skills training, conflict resolution) |
| Emotional skills development (e.g. emotion regulation, anger management) |
| Creative activity (e.g. arts, drama, music classes) |
| Physical activity (e.g. individual and team sports) |
| Signposting (e.g. leaflets, advice lines, websites) |
| Peer support (e.g. buddy systems) |
| Behaviour support (e.g. sanctions, behaviour management) |
| Designated space for well-being/mental health support (e.g. quiet rooms, time-out rooms, multi-sensory rooms etc.) |
| Infrastructure for extra-curricular activities (e.g. playground, music/drama room) |
| Individual therapy (e.g. counselling with a professional) |
| Group therapy (e.g. group counselling with a professional) |
| Mindfulness (e.g. meditation, breathing exercises, relaxation techniques) |
| Anti-Bullying programme |
| Risky health behaviour programme (e.g. substance abuse, smoking, drinking) |
| Mental health education (e.g. lessons on the topic of depression) |
|  |
| **For Parents/Carers:** (**not at all/ a little/ somewhat/ quite a lot/ very much)** |
| Information (e.g. leaflets, information evenings, advice lines) |
| Training (e.g. parenting programmes) |
| Counselling and support (e.g. family therapy, individual work with parent) |
|  |
| **For Staff:** (**Not at all/ A little/ somewhat/ quite a lot/ very much)** |
| Training and education (e.g. educating staff about mental health problems, identification) |
| Supervision and consultation (e.g. advice or supervision from mental health professional) |
| Counselling and support (e.g. provision for school staff to deal with stress and personal difficulties) |
| Well-being programme (e.g. provision for improvement of staff well-being, social activities) |
| *--------section break in online survey-----------* |
| In this section we enquire about the approaches your school takes to mental health and well-being provisions and the focus of such provision in terms of target groups of students. |
| **In your school what is the level of priority given to well-being and mental health provision?** |
| **(not a priority/ low priority/ medium priority/ high priority / essential)** |
|  |
| **In supporting student well-being to what extent does your school focus on:** |
| (**not at all/ a little/ somewhat/ quite a lot/ very much)** |
| Children with already identified mental health problems |
| Children with learning disabilities |
| Children starting to develop problems |
| Preventing problems from arising |
| Pro-actively promoting well-being |
|  |
| **In supporting student well-being to what extent does your school focus on:** |
| (**not at all/ a little/ somewhat/ quite a lot/ very much)** |
| Individuals with specific problems |
| The class level |
| The whole school |
|  |
| **Is your school implementing any policy related to mental health and well being provision?**  Yes/No |
|  |
| **Which institution(s) were involved in creating the above mentioned policy? (Please tick all that apply.)**  National government (in cooperation with relevant institutions and agencies) |
| Local government (in cooperation with relevant institutions and agencies) |
| School |
| Parents (or parent representatives) |
| Students (or student representatives) |
| Other (please specify) |
|  |
| **In supporting student well-being to what extent does your school focus on:** |
| (**not at all/ a little/ somewhat/ quite a lot/ very much)** |
| Individuals with specific problems |
| The class level |
| The whole school |
| *--------section break in online survey-----------* |
| In this section we ask about which professionals are typically involved in mental health and well-being provision in your school. |
|  |
| **Which of the following professionals are involved in mental health/well-being support in your school? (Please tick all that apply)** |
| School nurse |
| School psychologist |
| Learning support/ SEN support |
| Social worker |
| Clinical psychologist/psychiatrist |
| Other ____________ (please specify) |
| *--------section break in online survey-----------* |
| Thank you for completing this questionnaire. |
| For more information on the study please contact [Europeanschoolsurvey@gmail.com](mailto:Europeanschoolsurvey@gmail.com). |
|  |
| **Please indicate job role(s) of everyone involved in answering this survey. Choose as many as appropriate:** |
| Head Teacher |
| Deputy Head |
| Teacher |
| Teaching Assistant |
| Special Needs Coordinator |
| Administrator |
| School Psychologist |
| School Counsellor |
| School Nurse |
| Other ___________ (please specify) |
|  |
|  |
